# Comment on “A Unique Case of Cardiac Arrest following K2 Abuse”

**DOI:** 10.1155/2015/739149

**Published:** 2015-03-26

**Authors:** Joachim Alexandre, Danièle Debruyne, Antoine Coquerel, Reynald Le Boisselier

**Affiliations:** ^1^Department of Cardiology, CHU de Caen, 14000 Caen, France; ^2^Department of Pharmacology, CHU de Caen, 14000 Caen, France; ^3^EA 4650 Signalisation, Électrophysiologie et Imagerie des Lésions d'Ischémie-Reperfusion Myocardique, Université de Caen Basse-Normandie, 14000 Caen, France; ^4^Medical School, Université de Caen Basse-Normandie, 14000 Caen, France; ^5^Centre d'Évaluation et d'Information sur la Pharmacodépendance-Addictovigilance, CHU de Caen, 14000 Caen, France; ^6^Inserm U 1075 COMETE, Université de Caen Basse-Normandie, 14000 Caen, France

We appreciated reading the paper by Ibrahim et al. [1] describing the impact of K2 abuse on sudden cardiac death (SCD). This paper, although it provides interesting information about synthetic cannabinoid (SC), it also presents several limitations. Notably, the authors formally connected the K2 abuse and the SCD. In contrast to Ibrahim et al., we think that the SCD is mainly related to the cardiac history of the patient. Substantial evidence supports our point of view.

First, the patient had a past cardiological history with a previous myocardial infarction (MI) and four-vessel coronary artery bypass graft 10 years ago. Authors did not specify where the MI was located but we can suppose, from the electrocardiogram (ECG) in sinus rhythm, that it was an inferior MI (Q wave in inferior derivations). Moreover, authors did not specify the exact coronary lesions in native vessels. It would be interesting to know if one vessel (right coronary artery) was occluded. All these elements are important because it is well established that most of SCD cases in coronary artery disease patients occur on previous myocardial scar (which is the consequence of a coronary artery occlusion and results in the presence of Q wave in a coronary artery territory) and typically 10 years after MI [2–4]. Another element that supports our hypothesis is that authors declare that “the cardiac arrest was not associated with an acute coronary occlusion” [1] which is largely in favor of our theory. So it seems more reasonable to think that cardiac arrest is the consequence of a malignant reentrant ventricular arrhythmia than K2 abuse [2, 3].

It seems also difficult to conclude a myocardial necrosis based on moderate troponin T elevation (0.632). It would be helpful to specify the laboratory standards for the troponin T. The moderate elevation seems more likely due to the resuscitation (drugs, defibrillation, cardiac massage…) than a real myocardial necrosis which usually leads to major troponin T elevations [5].

Second, we have serious concerns regarding some cardiological interpretation of this observation. In fact, authors interpreted the ECG on admission [1] as a sinus tachycardia. It is wrong. This ECG shows a typical atrial flutter with a 2/1 ventricular conduction. We can recognize the atrial flutter with a regular and typical atrial activation near 240/min, uniform and continuous in all leads, with the typical “sawtooth” pattern in the inferior leads (manifested by a notching of the end of the QRS complex, clearly visible in V1 derivation and disappears during sinus rhythm in the second ECG presented by Ibrahim et al.). There is a classically 2 : 1 ventricular response [6]. The very long PR interval observed during tachycardia and absent in sinus rhythm also indicated that the rhythm cannot be sinusal in the first ECG. Atrial flutter following a cardiac arrest is classical when using drugs such as epinephrine or adrenaline and disappears most frequently in few hours or a day [7].

Regarding ECGs, we agree that cannabinoids have an impact on several ion channels but it seems difficult to directly link these properties to ECG changes, especially since the patient already has changes on his baseline ECG due to his coronary artery disease (and possibly antiarrhythmic drugs).

Third, evidence supports that cannabis is now a well-known trigger for MI [8, 9]. However, the challenge remains to link clearly the cardiac event to cannabinoids consumption and case reports are most often published without a clear etiology, with conclusions which are therefore most speculative. Conversely, the SC impact on serious cardiac events remains to date unknown, even if there are serious concerns [10–15]. The increasing use of SC as drug of abuse and their consequences in terms of public health have, therefore, to be closely monitored, especially considering pharmacological properties as the higher affinity for cannabinoid receptors than natural cannabinoid delta-9 THC resulting in higher pharmacological activity, early demonstrated with first SC [16, 17]. This present case report by Ibrahim et al. is therefore consistent with previously published reports but, to our knowledge, cannot reliably connect SC and SCD.

In conclusion, there are several evidences [10–15] to think that cannabis and SC can lead to serious cardiac disorders but we think that this case report is speculative rather than affirmative (because of the study limitations described above) and therefore, conclusions should be more modulated.

## References

[1] Ibrahim S., Al-Saffar F., Wannenburg T. (2014). A unique case of cardiac arrest following K2 abuse. *Case Reports in Cardiology*.

[2] Alexandre J., Saloux E., Dugué A. E. (2013). Scar extent evaluated by late gadolinium enhancement CMR: a powerful predictor of long term appropriate ICD therapy in patients with coronary artery disease. *Journal of Cardiovascular Magnetic Resonance*.

[3] Alexandre J., Saloux E., Lebon A. (2014). Scar extent as a predictive factor of ventricular tachycardia cycle length after myocardial infarction: implications for implantable cardioverter-defibrillator programming optimization. *Europace*.

[4] Haqqani H. M., Marchlinski F. E. (2009). Electrophysiologic substrate underlying postinfarction ventricular tachycardia: Characterization and role in catheter ablation. *Heart Rhythm*.

[5] Reichlin T., Hochholzer W., Bassetti S. (2009). Early diagnosis of myocardial infarction with sensitive cardiac troponin assays. *The New England Journal of Medicine*.

[6] Saoudi N., Cosío F., Waldo A. (2001). A classification of atrial flutter and regular atrial tachycardia according to electrophysiological mechanisms and anatomical bases: a statement from a joint expert group from the working group of arrhythmias of the European society of cardiology and the North American society of pacing and electrophysiology. *European Heart Journal*.

[7] Kaumann A. J., Sanders L. (1993). Both *β*1- and *β*2-adrenoceptors mediate catecholamine-evoked arrhythmias in isolated human right atrium. *Naunyn-Schmiedeberg's Archives of Pharmacology*.

[8] Nawrot T. S., Perez L., Künzli N., Munters E., Nemery B. (2011). Public health importance of triggers of myocardial infarction: a comparative risk assessment. *The Lancet*.

[9] Mittleman M. A., Maclure M., Sherwood J. B. (1995). Triggering of acute myocardial infarction onset by episodes of anger. Determinants of Myocardial Infarction Onset Study Investigators. *Circulation*.

[10] Mir A., Obafemi A., Young A., Kane C. (2011). Myocardial infarction associated with use of the synthetic cannabinoid K2. *Pediatrics*.

[11] Jouanjus E., Lapeyre-Mestre M., Micallef J., French Association of the Regional Abuse and Dependence Monitoring Centres (CEIP-A) Working Group on Cannabis Complications (2014). Cannabis use: signal of increasing risk of serious cardiovascular disorders. *American Heart Association Journals*.

[12] Tse R., Kodur S., Squires B., Collins N. (2014). Sudden cardiac death complicating acute myocardial infarction following synthetic cannabinoid use. *Internal Medicine Journal*.

[13] Gunawardena M. D. V. M., Rajapakse S., Herath J., Amarasena N. (2014). Myocardial infarction following cannabis induced coronary vasospasm. *BMJ Case Reports*.

[14] Hodcroft C. J., Rossiter M. C., Buch A. N. (2014). Cannabis-associated myocardial infarction in a young man with normal coronary arteries. *The Journal of Emergency Medicine*.

[15] McKeever R. G., Vearrier D., Jacobs D., LaSala G., Okaneku J., Greenberg M. I. (2014). K2—not the spice of life; synthetic cannabinoids and st elevation myocardial infarction: a case report. *Journal of Medical Toxicology*.

[16] Huffman J. W., Zengin G., Wu M.-J. (2005). Structure-activity relationships for 1-alkyl-3-(1-naphthoyl)indoles at the cannabinoid CB(1) and CB(2) receptors: steric and electronic effects of naphthoyl substituents. New highly selective CB(2) receptor agonists. *Bioorganic and Medicinal Chemistry*.

[17] Weissman A., Milne G. M., Melvin L. S. (1982). Cannabimimetic activity from CP-47,497, a derivative of 3-phenylcyclohexanol. *Journal of Pharmacology and Experimental Therapeutics*.

